# TAWFN: a deep learning framework for protein function prediction

**DOI:** 10.1093/bioinformatics/btae571

**Published:** 2024-09-23

**Authors:** Lu Meng, Xiaoran Wang

**Affiliations:** College of Information Science and Engineering, Northeastern University, Shenyang, Liaoning, 110000, China; College of Information Science and Engineering, Northeastern University, Shenyang, Liaoning, 110000, China

## Abstract

**Motivation:**

Proteins play pivotal roles in biological systems, and precise prediction of their functions is indispensable for practical applications. Despite the surge in protein sequence data facilitated by high-throughput techniques, unraveling the exact functionalities of proteins still demands considerable time and resources. Currently, numerous methods rely on protein sequences for prediction, while methods targeting protein structures are scarce, often employing convolutional neural networks (CNN) or graph convolutional networks (GCNs) individually.

**Results:**

To address these challenges, our approach starts from protein structures and proposes a method that combines CNN and GCN into a unified framework called the two-model adaptive weight fusion network (TAWFN) for protein function prediction. First, amino acid contact maps and sequences are extracted from the protein structure. Then, the sequence is used to generate one-hot encoded features and deep semantic features. These features, along with the constructed graph, are fed into the adaptive graph convolutional networks (AGCN) module and the multi-layer convolutional neural network (MCNN) module as needed, resulting in preliminary classification outcomes. Finally, the preliminary classification results are inputted into the adaptive weight computation network, where adaptive weights are calculated to fuse the initial predictions from both networks, yielding the final prediction result. To evaluate the effectiveness of our method, experiments were conducted on the PDBset and AFset datasets. For molecular function, biological process, and cellular component tasks, TAWFN achieved area under the precision-recall curve (AUPR) values of 0.718, 0.385, and 0.488 respectively, with corresponding *F*_max_ scores of 0.762, 0.628, and 0.693, and *S*_min_ scores of 0.326, 0.483, and 0.454. The experimental results demonstrate that TAWFN exhibits promising performance, outperforming existing methods.

**Availability and implementation:**

The TAWFN source code can be found at: https://github.com/ss0830/TAWFN.

## 1 Introduction

Proteins’ roles within biological organisms are indispensable, encompassing vital life processes such as signal transduction, catalyzing metabolic reactions, and maintaining cellular structures. Accurately identifying protein functions allows for a deeper understanding of disease mechanisms and holds promise for discovering new therapeutic targets (Eisenberg *et al.* 2000). Despite the surge in protein sequence data facilitated by high-throughput technologies, determining the precise functions of proteins still demands significant time and resources. For instance, when performing a BLAST (Altschul *et al.* 1997) sequence alignment for a mouse protein LYPA2_MOUSE (Uniprot Symbol: Q9WTL7) with a sequence length of 231, it took 39 000 ms. If such sequence comparisons are extended to millions of protein sequences followed by analysis and annotation, significantly more time would be required. Therefore, many protein functions remain incompletely resolved. Developing an accurate and efficient protein function prediction method is thus of paramount importance. Protein function prediction involves analyzing a given protein sequence or related information to infer the gene ontology (GO) terms it might possess. These GO terms consist of three categories: biological process (BP), molecular function (MF), and cellular component (CC). Using GO:0031981 in the CC category as an example, it represents the volume surrounded by the nuclear membrane. Its ancestral graph is illustrated in Supplementary Fig. S1. Each term in every ontology category can be represented as a directed acyclic graph, where parent nodes of broader functional terms point to child nodes of more specific functional terms. Current methods for predicting protein functions focus on three main areas: protein sequences, protein structures, and protein–protein interaction networks.

For protein sequences, Kulmanov *et al.* used convolutional methods on groups of three amino acids (Kulmanov *et al.* 2018) and one-hot encoding with different convolutional kernels (Kulmanov and Hoehndorf 2020), incorporating BLAST sequence similarity (Altschul *et al.* 1997). One-hot encoding is sparse, so protein language models like ESM (Rives *et al.* 2021), ProtTrans (Elnaggar *et al.* 2021), and UDSMProt (Strodthoff *et al.* 2020) are used. Zhu *et al.* proposed ATGO (Zhu *et al.* 2022), which uses a pre-trained language model for protein sequences embedded within a triple neural network architecture to predict protein functions. Yuan’s SPROF-GO (Yuan *et al.* 2023) and Kulmanov’s DeepGO-SE (Kulmanov *et al.* 2024) both use sequence features generated by pre-trained protein language models to enhance the prediction performance of GO terms.

Biomedical literature is also used for prediction. DeepText2GO predicts functions using textual representation and sequence information (You *et al.* 2018), while DeepAF uses ESM for sequence feature extraction (Zhao *et al.* 2023). However, not all proteins have literature available.

For protein–protein interaction networks, methods predict functions based on connected proteins sharing functions (Oliver 2020). NetGO transfers known functions of interacting proteins to the target protein (You *et al.* 2019), with NetGO 2.0 incorporating literature and sequence information (Yao *et al.* 2021). These methods depend on functionally annotated proteins.

Structure-based methods use protein structures as inputs for graph neural networks (GNN) like DeepFRI, using structural models from PDB (Berman *et al.* 2020) and SWISS-MODEL (Waterhouse *et al.* 2018). Lai and Xu developed the GAT-GO method (Lai and Xu 2022), a graph attention network approach that significantly enhances protein function prediction by leveraging predicted structural information and protein sequence embeddings. AlphaFold2 is essential for predicting structures. EGNN combines embeddings from pre-trained models with graph representations from AlphaFold2 (Boadu *et al.* 2023). HEAL uses AlphaFold2-predicted structures and the PDB database, employing Transformers for prediction (Gu *et al.* 2023).

Currently, using CNN or graph convolutional network (GCN) alone presents the following issues:

1. Information loss can occur when using CNNs because their local receptive fields may not capture the global structure of proteins. If key features are spread over a large area, CNNs might miss them by only focusing on local regions. Similarly, with GCNs, if the protein structure has complex relationships that the graph convolutional layers cannot fully capture, it can result in incomplete representation of the protein’s features.
2. GCNs are more suitable for handling graph-structured data, while CNNs are better for sequence data. Using only one of these networks may not fully capture the diverse information in proteins, leading to limitations in feature representation. When protein structures include both sequence and graph information, relying on just one type of network cannot fully utilize these different sources, limiting a complete understanding of the protein structures.

To address these issues, we propose a novel protein function prediction method called the two-model adaptive weight fusion network. Our approach integrates CNN and GCN, leveraging both protein structure and protein language models. Protein structures are processed to obtain corresponding protein sequences.

Our primary contributions can be summarized as follows:

- In the aspect of GCNs, we construct a graph input network using the processed protein sequence features and the protein contact map. We employ GCN encoders to capture short-range information and introduce Transformers to capture long-range information. To better understand the topological semantics, we utilize attention mechanisms to generate graph representations.
- In the convolutional network aspect, we use the processed protein sequence features as input. We employ a multi-layer convolutional encoder, where multiple convolutional layers are cascaded. Additionally, we integrate a feature pyramid structure with a multi-scale deep feature extractor to capture local features. Furthermore, we introduce a multi-head attention mechanism to capture long-range dependencies between multi-scale local features.
- By employing adaptive weight computation, we fuse the preliminary prediction results from both networks to obtain the final prediction outcome.
- We conducted extensive experiments comparing TAWFN with baseline methods, including Blast (Altschul *et al.* 1997), FunFam (Das *et al.* 2015), DeepGO (Kulmanov *et al.* 2018), DeepGOPlus (Kulmanov and Hoehndorf 2020), DeepFRI (Gligorijević *et al.* 2021), GAT-GO method (Lai and Xu 2022), ATGO (Zhu *et al.* 2022), SPROF-GO (Yuan *et al.* 2023), DeepGO-SE (Kulmanov *et al.* 2024), and HEAL (Gu *et al.* 2023). The results demonstrate that our performance surpasses other state-of-the-art methods, such as DeepFRI (Gligorijević *et al.* 2021) and HEAL (Gu *et al.* 2023). Our model also exhibits outstanding generality and excellent interpretability, indicating that combining both approaches leads to improved effectiveness.

## 2 Materials and methods

### 2.1 Overview

**Figure 1.**
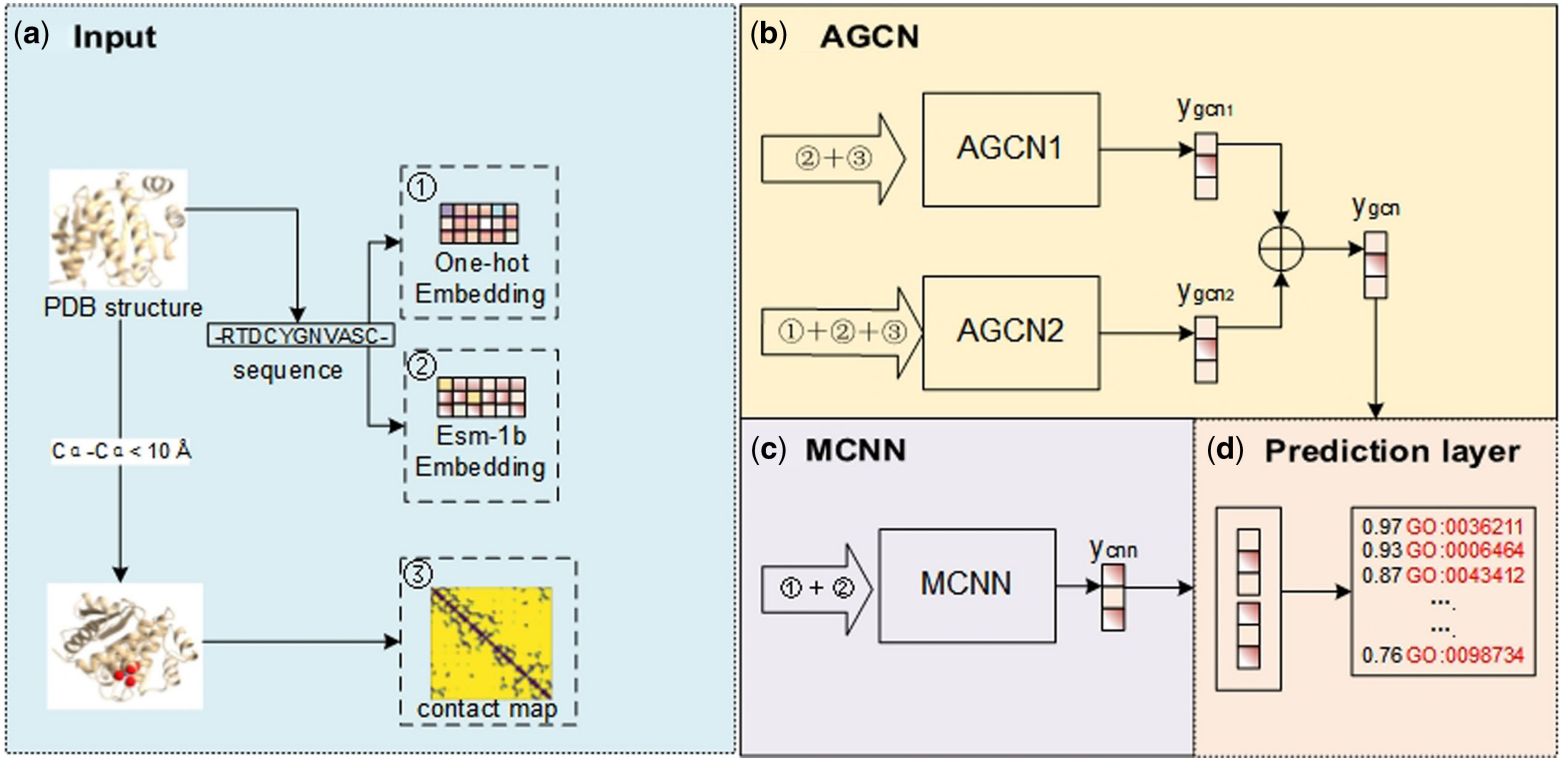
The TAWFN model graph. The model consists of four modules: (a) input data generation module: this module generates protein contact maps and sequence encoding features, including ESM-1b encoding and one-hot encoding. (b) AGCN module based on GCN: this module includes two sub-modules, ${\mathrm{AGCN}}_{1}$ and ${\mathrm{AGCN}}_{2}$. Both sub-modules share the same AGCN network while processing different inputs. It produces the preliminary prediction results, $y_{\mathrm{gcn}}$. (c) MCNN module based on CNN: this module generates preliminary prediction results, $y_{\mathrm{cnn}}$. (d) Adaptive fusion module: this module fuses the two preliminary prediction results, $y_{\mathrm{gcn}}$ and $y_{\mathrm{cnn}}$, through computation to generate the final prediction scores.

The structure of TAWFN, as shown in Fig. 1, primarily consists of four modules: Input data generation module, adaptive GCNs module, multi-layer convolutional neural network module, and adaptive fusion module. We use the input data generation module to construct the inputs, then train the inputs using AGCN and MCNN. Finally, we fuse the results from both using an adaptive fusion network.

### 2.2 Input data generation

#### 2.2.1 Sequence features

In this method, two types of sequence features for proteins are utilized. One is obtained through the ESM-1b language model (Rives *et al.* 2021) to acquire semantic encoding, while the other is one-hot encoding. (i) ESM-1b encoding utilizes the ESM-1b model (Rives *et al.* 2021) for protein sequence encoding. ESM-1b is a pre-trained language model based on large-scale protein sequences, similar to the BERT model (Kenton and Toutanova 2019) in the field of natural language processing. ESM-1b model is pre-trained on a vast protein database to learn semantic information and patterns of protein sequences, enabling the mapping of protein sequences into a high-dimensional semantic space. In this method, a matrix of size *L* × 1280 is obtained, where *L* represents the sequence length, for subsequent input. (ii) For the protein sequences obtained from structures, they are encoded using one-hot encoding. In this representation, each amino acid is encoded as a specific number. For example, for a protein sequence of length *L*, it is encoded into a 1D vector of length *L*, where each amino acid is replaced with a numerical identifier ranging from 0 to 20, representing the 20 common amino acids and one for a missing position. The missing position refers to an unknown amino acid not belonging to the 20 common amino acids at a specific sequence position (Liu *et al.* 2016). This situation is considered during experiments. This encoding method retains the sequence information between amino acids. To combine it with ESM-1b encoding and extend it to the same dimension, an embedding operation is utilized to transform it into a 2D matrix of size *L* × 96, facilitating subsequent combinations.

#### 2.2.2 Construction maps

Based on the protein structure, contact maps are constructed, representing the distances between all pairs of amino acid residues in the protein structure. The protein contact maps are represented as a 2D matrix. We employ a distance threshold method, where carbon atoms in the protein are connected if the distance between them is within a specified distance threshold. Assuming there are two carbon atoms $\left(u,\;v\right)$, if $\left|u-v\right|\;\geq \;\theta$, i.e. it is less than the set threshold, then it is considered that there is no connection between the two carbon atoms. Conversely, if $\left|u-v\right|\;\geq \;\theta$, i.e. it is greater than or equal to the set threshold, then it is considered that there is an edge connection between the two carbon atoms, where θ is the distance threshold. In this method, the threshold θ is set to 10 Å. Finally, the edge index $E_{\mathrm{index}}\in R^{2\times E}$ corresponding to the protein structure is obtained, where *E* represents the number of edges.

### 2.3 Adaptive GCNs

**Figure 2.**
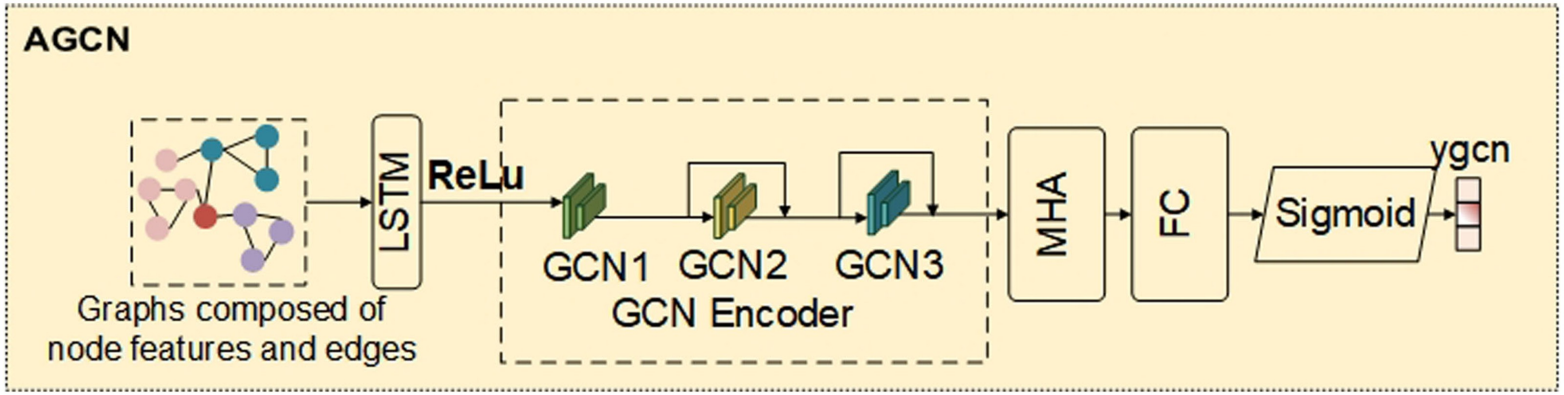
AGCN structure diagram. The input is a graph composed of node features and edge relationships. ${\mathrm{GCN}}_{1},{\mathrm{GCN}}_{2}{,\;\mathrm{GCN}}_{3}$ represent three layers of GCN, which have the same structure. MHA is the multi-head attention module proposed in our method, and $y_{\mathrm{gcn}}$ is the prediction output.

Protein structure can be represented as a graph, where amino acid residues represent nodes, and the contacts between residues represent edges. For the AGCN module, there are two sub-modules: ${\mathrm{AGCN}}_{1}$ and ${\mathrm{AGCN}}_{2}$. In ${\mathrm{AGCN}}_{1}$, the input consists of node features obtained from ESM-1b encoding and the relationships between residues obtained from the protein contact map, forming a graph. Since ESM-1b encoding carries more protein information compared to one-hot encoding, combining them weakens the information in ESM-1b. Therefore, in experiments, only the ESM-1b encoding is initially inputted as node features. Considering comprehensive feature information, in ${\mathrm{AGCN}}_{2}$, the input consists of node features obtained from the combination of one-hot encoding and ESM-1b encoding, along with the relationships between residues obtained from the protein contact map, forming a graph. The network structure of ${\mathrm{AGCN}}_{1}$ and ${\mathrm{AGCN}}_{2}$ is the same, with only different inputs during experiments. The AGCN structure is illustrated in Fig. 2.

After inputting into AGCN, the node features first undergo processing through the LSTM module. LSTM is effective in handling sequential data and can capture long-range dependencies within the sequence, thereby extracting richer feature representations. Afterwards, the node features are passed through three layers of GCN encoders, with each layer having the same structure. The node features and relationships between nodes are inputted into the GCN encoder. In each layer, the features of each node are updated iteratively by computing the features of adjacent nodes (nodes connected by edges to the current node). The computation formula of GCN is shown in Equation (1)
 

(1) $$H^{l+1}=\mathrm{ReLU}\left({\tilde{D}}^{-0.5}\tilde{A}{\tilde{D}}^{-0.5}H^{\left(l\right)}W^{\left(l\right)}\right)\;,$$

where $H^{\left(l\right)}$ represents the node feature matrix of the *l*th layer, $W^{\left(l\right)}$ is the weight matrix of the *l*th layer, $\tilde{A}$ = *A* + *I* is the adjacency matrix *A* added with self-connections (plus the identity matrix), $\tilde{D\;}$is the degree matrix of $\tilde{A}$, and ReLU(.) is the non-linear activation function. After passing through the GCN network layers, sufficient local geometric information is embedded.

**Figure 3.**
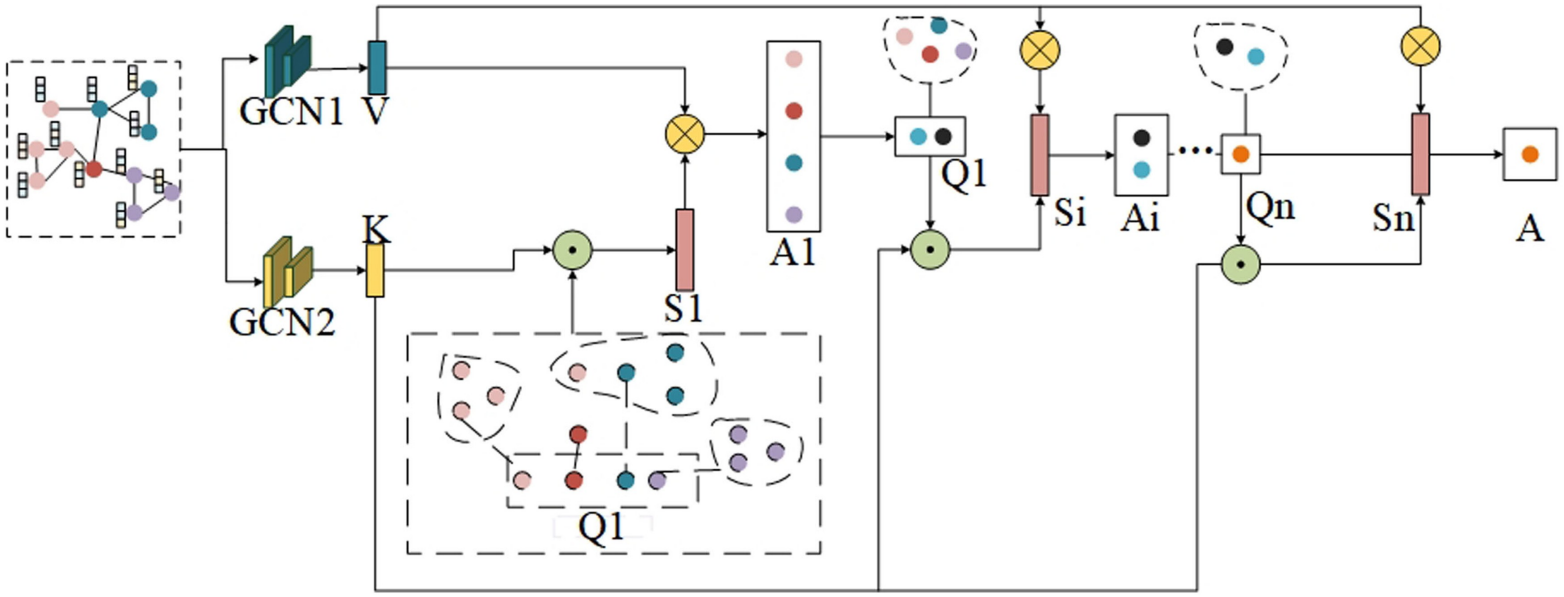
The structure of the multi-head attention (MHA). The input consists of the graph representation after being processed by the GCN encoder. The input undergoes GCN transformation, where a single-layer GCN, identical to the one used in the GCN encoder, is employed. This process generates key vectors (*K*) and value vectors (*V*) for the attention mechanism. Additionally, randomly generated features for *m* nodes serve as query vectors (*Q*), which are then split into multiple heads along with *K* and *V* for parallel computation. Each head computes the dot product between *K* and *Q*, resulting in a set of weight vectors (*S*). Multiplying *S* by *V* yields the weighted representations of *m* nodes. This operation is repeated until the last attention mechanism iteration is reached, at which point the entire node features are computed into a single node feature representation, effectively setting *m* to 1.

In this approach, inspired by the HEAL (Gu *et al.* 2023) method, a Transformer (Baek *et al.* 2021) model is introduced, similar to the multi-head attention mechanism, to capture long-range features using learnable node features. In this mechanism, features for *m* nodes are randomly generated, and these generated node features are treated as query vectors after linear transformation. Simultaneously, the input node features are transformed through GCN and regarded as key (*K*) and value (*V*) vectors, respectively. The structure diagram of the multi-head attention mechanism (MHA) is illustrated in Fig. 3.

Splitting *K*, *Q*, and *V* into the specified number of heads, we calculate a set of weights by computing the similarity between the query vector $Q_{i}\in R^{m\times D}$ and the key vector $K_{i}\in R^{L\times D}$. This yields a set of weights, as shown in Equation (2), which are then used to weight the value vectors to obtain $\Psi_{i}\in R^{m\times D}$.

(2) $$\Psi_{i}=\mathrm{softmax}\left(\frac{Q_{i}\cdot {K_{i}}^{⊤}}{\sqrt{D}}\right)\cdot V_{i},$$

where $Q_{i}$ and $K_{i}$ represent the query vector and key vector of the current head, respectively, their dot product is divided by a scaling factor. Then, the softmax function is applied to compute the attention weights, which are multiplied by the current value vector $V_{i}\in R^{L\times D}$ to calculate the weighted feature.

This attention mechanism allows the model to focus on node features most relevant to the query. Finally, all the weighted features computed by each head are concatenated together, as shown in Equation (3), to obtain $A\in R^{m\times D}$
 

(3) $$A=\left[\Psi_{1},\Psi_{2}\ldots ,\Psi_{H}\right].$$

According to the training settings, the value of *m* is continually updated. When processing for the last time, *m* is set to 1, meaning that all node features of a protein sequence are aggregated into a single node feature representation, similar to setting the parameter to 1 in adaptive pooling layers, resulting in a single value. The entire process uses the multi-head attention mechanism to compute attention weights for node features and achieves a pooling-like operation, aggregating node features into a new representation. This operation aims to capture global features of the entire protein structure.

The results obtained from ${\mathrm{AGCN}}_{1}$ and ${\mathrm{AGCN}}_{2}$ are merged through adaptive fusion to obtain the final result of AGCN, following the calculation process described in Equation (4)
 

(4) $$A_{\mathrm{final}}=\alpha \cdot A_{\mathrm{esm}}+\left(1-\alpha \right)\cdot A_{\mathrm{esm}-\mathrm{onehot}}.$$

As shown in Equation (5), the result is fed into a fully connected layer, followed by ReLU activation, dropout layer, and sigmoid activation, to obtain the preliminary classification result of AGCN

(5) $${\hat{y}}_{\mathrm{gcn}}=\mathrm{Sigmoid}(\mathrm{ReLu}\left(W\left(A_{\mathrm{final}})\right)\right),$$

where ${\hat{y}}_{\mathrm{gcn}}\in R^{C}$ represents the predicted result of AGCN, *C* is the number of GO terms, and *W* denotes the weights of the fully connected layer, where ${\hat{y}}_{\mathrm{gcn}}$ each element represents the positive probability of each GO term.

During training, we utilize the cross-entropy loss function, as depicted in Equation (6). The cross-entropy loss function is a commonly used loss function for multi-class classification problems. In protein function prediction, there are typically multiple different functional categories involved, making the cross-entropy loss function well-suited for this scenario

(6) $$L=-\sum_{m=1}^{M}\sum_{n=1}^{C}(y_{mn}l\mathrm{og}\left({\hat{y}}_{mn}\right)+\left(1-y_{mn}\right)l\mathrm{og}\left(1-{\hat{y}}_{mn}\right),$$

where *M* represents the number of training sequences, *C* denotes the number of GO terms, ${\hat{y}}_{mn}\;\in$ [0, 1] represents the predicted probability, and $y_{mn}\;\in$ {0, 1} denotes the ground truth for the *m*th sequence along the *n*th GO term.

### 2.4 Multi-layer convolutional neural network

**Figure 4.**
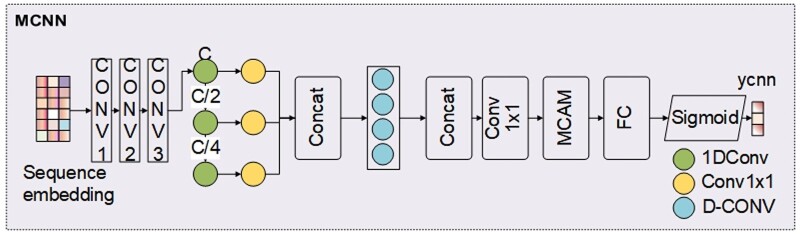
The architecture of MCNN. The input to MCNN is the encoded node features. MCAM represents the proposed multi-channel attention mechanism, and $y_{\mathrm{cnn}}$ denotes the prediction output.

The MCNN architecture, as shown in Fig. 4, is inspired by MMSMAPlus (Wang *et al.* 2023). MCNN consists of multiple layers of cascaded convolutional encoders that process the input sequence features. The input to MCNN is the node features composed of one-hot and ESM-1b. It starts with a three-layer cascaded convolutional encoder to extract local feature information. Then, the feature pyramid structure is integrated into the method to ensure scale invariance. After obtaining local features, further global features are extracted by introducing a multi-head attention mechanism (Yang *et al.* 2021).

The three layers of 1D convolution in MCNN serve to extract and normalize the input protein sequence features. 1D convolution operates in a highly parallel manner, sliding convolutional kernels along the sequence to capture local features at different positions within the protein sequence. Following this, batch normalization normalizes the convolutional output, enhancing the stability and convergence speed of the MCNN model, thereby accelerating the training process.

Capturing more local features improves protein function prediction accuracy (Zhang *et al.* 2021). However, using too many convolution filters can make the network too complex and increase computation without improving performance (Kulmanov *et al.* 2018, Kulmanov and Hoehndorf 2020, Zhang *et al.* 2021). Following the MMSMAPlus approach (Wang *et al.* 2023), a feature pyramid is used to extract multi-scale features, as different scales offer valuable information (Bdaneshvar 2017). Convolutional layers are cascaded, and features at three scales (512, 256, and 128) are upsampled, expanded to the same size, and then combined. Depthwise separable convolution and 1 × 1 convolution are then used to further extract multi-scale features.

**Figure 5.**
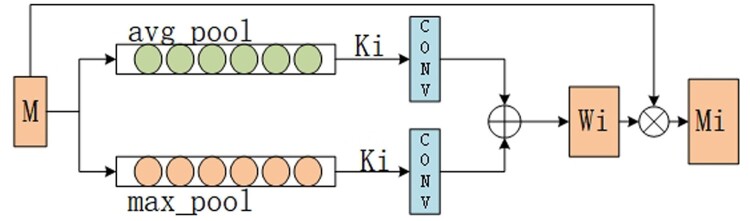
The structure of the CAM.

To capture long-range dependencies, similar to the attention mechanism in AGCN, MCNN also employs a multi-head attention mechanism, but it focuses more on extracting global information from the input sequence. We refer to this as the multi-head channel attention mechanism (MCAM), allowing each head to focus on different important features within the sequence and integrate these features through weighted fusion. The individual channel attention mechanism (CAM) structure for each head in MCAM is illustrated in Fig. 5.

After processing the features, we perform both average pooling and max pooling to obtain $M_{\mathrm{avg}}$ and $M_{\max}$, respectively. Following the idea of channel attention mechanism, $M_{\mathrm{avg}}$ and $M_{\max}$ are separately input into a shared 1D convolution to derive ${M_{\mathrm{avg}}^{\prime }}^{}$ and $M_{\max}^{\prime }$. These are then summed, passed through a sigmoid function to map the results to the range [0, 1], serving as the attention weights for each head, resulting in corresponding weight values $W_{i}$. Next, we multiply the original input feature *M* by the calculated attention weights $W_{i}$ to obtain the processed feature $M_{i}$. To better capture features at different scales, we employ the idea of multi-head attention mechanism, where each $K_{i}$ adopts a different value, representing different convolution kernel sizes. Finally, the obtained $M_{i}$ is used to automatically calculate the weights during training, as shown in Equation (7)
 

(7) $$M_{\mathrm{final}}=\sum_{h=1}^{h}\alpha_{i}\cdot M_{i}\;s.t.\alpha^{T}1=1,\alpha \geq 0,$$

where $M_{\mathrm{final}}$ represents the feature result after processing with MCAM, *h* denotes the number of heads, $\alpha_{i}$ represents the weight of the *i*th head, and $M_{i}$ represents the feature after the *i*th channel attention mechanism.

In this process, we leverage multiple channel attention modules to obtain results for each head. It can weight information at different levels, generating multi-head attention representations. This highly parallel structure can effectively preserve crucial information while fully exploring the rich semantics of the input data.

During training, we also use the cross-entropy loss function [Equation (6)] as the loss function for MCNN. The output of MCNN is similarly passed through a fully connected layer, followed by ReLU activation, a dropout layer, and a sigmoid activation function, computed as shown in Equation (8)
 

(8) $${\hat{y}}_{\mathrm{cnn}}=\mathrm{Sigmoid}(\mathrm{ReLu}\left(W\left(M_{\mathrm{final}})\right)\right),$$

where *W* represents the weights of the fully connected layer, ${\hat{y}}_{\mathrm{cnn}}\in R^{C}$ denotes the prediction vector, and *C* is the number of GO terms. Each element of ${\hat{y}}_{\mathrm{cnn}}$ represents the positive probability of each GO term and is also the preliminary classification result of MCNN.

To obtain the final result, we combine the preliminary predictions of AGCN and MCNN through joint decision-making. Using adaptive weighting calculation according to Equation (9), we derive the best prediction result. This approach leverages the strengths of both GCN and CNN to thoroughly learn features

(9) $$\hat{f}=\alpha \cdot {\hat{y}}_{\mathrm{gcn}}+\left(1-\alpha \right)\cdot {\hat{y}}_{\mathrm{cnn}}.$$

Finally, we optimize the integrated prediction result using the cross-entropy loss function [Equation (6)] to obtain the final prediction result.

## 3 Experimental results

### 3.1 Dataset

We used the same dataset as HEAL (Gu *et al.* 2023), available at https://github.com/ZhonghuiGu/HEAL. The dataset includes 36 629 protein structures from the PDB database (PDBset) and 42 994 protein structures with GO terms from the AlphaFold protein structure database (AFset). The PDBset contains representative PDB chains with at least one functional annotation and high-resolution structures, divided into training, validation, and testing sets at an 8:1:1 ratio. Experimental structures were extracted from the PDB for each sequence, and protein graphs were constructed. GO term annotations were obtained from SIFTS (Dana *et al.* 2019) and UniProtKB, with each sequence labeled with 489 MF, 1943 BP, and 320 CC terms. The AFset consists of 42 427 sequences in the AFset training set and 567 sequences in the AFset test set. Any sequences with over 25% identity to those in the AFset training set or PDBset training set were removed from the AFset test set. Finally, 10% of the sequences were randomly selected from the AFset training set to form the validation set.

The sequences in the PDBset test set are grouped based on their homology, which is measured by comparing sequence similarities. We conduct experiments using different similarity thresholds to determine how similar two protein sequences are. The thresholds we use are 30%, 40%, 50%, 70%, and 95%, allowing us to study performance at various levels of sequence homology.

### 3.2 Model training

During training, we employed the Adam optimizer (Kingma and Ba 2015) to train the proposed method TAWFN. The learning rates for AGCN and MCNN were set to 0.0001 and 0.0005, respectively, with batch sizes of 48 and 64 and 100 epochs. The implementation was based on PyTorch and PyTorch Geometric libraries (Fey and Lenssen 2019). To prevent unnecessary training when no improvement is observed, we utilized early stopping criteria with a patience of five epochs. Training was conducted on an NVIDIA GeForce RTX 3090 24G GPU.

### 3.3 Evaluation metrics

We primarily used the metrics $F_{\max}$ (Radivojac *et al.* 2013), $S_{\min}$ (Clark and Radivojac 2013), and AUPR (Davis and Goadrich 2006) to evaluate the performance of our method. The $F_{\max}$ metric represents the maximum *F*-value calculated across all prediction thresholds. The $S_{\min}$ indicates the semantic distance between predicted and true annotations, considering the information content of each function. The AUPR is approximated using the trapezoidal rule to compute the area under the precision–recall curve, evaluating the model’s performance across different prediction thresholds. Lower $S_{\min}$ are preferable, while higher values for $F_{\max}$ and AUPR indicate better performance. Additional details on how to compute these metrics can be found in Supplementary material.

### 3.4 Comparison of TAWFN with other state-of-the-art methods

We compared our method with several baseline methods. We evaluated the performance of these methods on the PDBset test set across three gene ontology domains: MF, BP, and CC. The results are summarized in Table 1. TAWFN outperformed all other methods in all three gene ontology domains. TAWFN achieves AUPR scores of 0.718, 0.385, 0.488, $F_{\max}$ scores of 0.762, 0.628, 0.693, and $S_{\min}$ scores of 0.326, 0.483, 0.454 on the MF, BP, and CC tasks, respectively. Additionally, TAWFN was tested against other methods on the CAFA3 test set, and the results can be found in Supplementary Table S6. These results surpass the performance of the best GCN-based method HEAL. This indicates that our proposed TAWFN, which combines GCN and CNN, can more comprehensively learn features within protein structures. Additionally, the MHA in AGCN effectively learns protein graph features through graph pooling, while the MCAM in MCNN captures global features of protein sequences. These factors contribute to the effectiveness of our method in protein function prediction.

**Table 1.** AUPR, *F*_max_, and *S*_min_ of different methods on PDBset test set.

Method	AUPR(↑)			$F_{\max}$ (↑)			$S_{\min}$ (↓)		
	MF	BP	CC	MF	BP	CC	MF	BP	CC
BLAST	0.136	0.067	0.096	0.326	0.336	0.443	0.643	0.662	0.632
FunFams	0.37	0.256	0.265	0.573	0.498	0.64	0.542	0.58	0.512
DeepGO	0.391	0.189	0.258	0.576	0.50	0.589	0.475	0.578	0.553
DeepFRI	0.495	0.265	0.274	0.627	0.546	0.617	0.432	0.543	0.530
GAT-GO	0.66	0.381	0.479	0.633	0.492	0.547	0.437	0.521	0.466
ATGO	0.708	0.249	0.306	0.76	0.318	**0.703**	0.336	0.600	0.539
SPROF-GO	0.606	0.209	0.307	0.750	0.454	0.627	0.336	0.562	0.512
DeepGO-SE	0.495	0.233	0.423	0.654	0.566	0.636	0.435	0.53	0.481
HEAL	0.661	0.339	0.435	0.733	0.613	0.673	0.357	0.499	0.475
TAWFN	**0.718**	**0.385**	**0.488**	**0.762**	**0.628**	0.693	**0.326**	**0.483**	**0.454**

The bold texts indicate the maximum values in the table.

### 3.5 Ablation study

For the MCNN and AGCN, we designed ablation experiments to validate the effectiveness of their combination, and verified the role of LSTM in AGCN. The results are shown in Table 2. It can be observed that the performance of MCNN is better than that of AGCN, indicating that the global information generated by MCAM in MCNN is beneficial for protein prediction. Moreover, when both MCNN and AGCN are combined in the network, the performance is better than when each module predicts separately. This suggests that the combination not only improves the learning of features from both local and global perspectives but also focuses on details. Overall, our proposed method has an enhancing effect on protein function prediction performance.

**Table 2.** Validating the impact of MCNN and AGCN on models on PDBset.

AGCN (without LSTM)	AGCN	MCNN	AUPR(↑)			$F_{\max}$ (↑)			$S_{\min}$ (↓)		
			MF	BP	CC	MF	BP	CC	MF	BP	CC
√			0.667	0.322	0.435	0.727	0.601	0.670	0.357	0.508	0.470
		√	0.700	0.365	0.46	0.747	0.608	0.686	0.344	0.499	0.463
	√		0.663	0.339	0.468	0.733	0.613	0.673	0.357	0.499	0.475
	√	√	**0.718**	**0.385**	**0.488**	**0.762**	**0.628**	**0.693**	**0.326**	**0.483**	**0.454**

The bold texts indicate the maximum values in the table.

### 3.6 Impact of other variables

We compared different configurations by varying the number of attention heads in MCNN and the number of GCN layers in AGCN. Specifically, we tested the effects of different numbers of attention heads and GCN layers on model performance. Detailed results are presented in Supplementary Tables S1 and S2. The results show that simply increasing the number of attention heads in MCNN does not always improve performance. While more heads might enhance the model’s capacity, having too many can introduce redundant information, potentially decreasing prediction accuracy. Therefore, choosing the right number of heads is crucial. Similarly, adding more GCN layers in AGCN can enhance the model’s capability but also increases computational costs and may lead to overfitting, which reduces overall performance. Balancing model complexity and computational efficiency is essential. Based on these findings, we selected the optimal configurations: four attention heads for MCNN and three GCN layers for AGCN. This combination provides a good balance between performance and computational efficiency.

### 3.7 TAWFN enhancements

#### 3.7.1 TAWFN performance at different thresholds

To assess the generalization ability of TAWFN, we evaluated it on the PDBset test set, which contains sequences with different levels of homogeneity compared to the training set. The sequence similarity thresholds were set to 30%, 40%, 50%, 70%, and 95%. We conducted experiments comparing TAWFN with DeepGO (Kulmanov *et al.* 2018), DeepFRI (Gligorijević *et al.* 2021), and HEAL (Gu *et al.* 2023) (Supplementary Table S3 through S5).

Our method demonstrates superior performance across all five thresholds for MF, BP, and CC, outperforming other methods in terms of evaluation metrics. The combination of AGCN and MCNN results through adaptive weighting calculation yielded the best performance, indicating a better capture of protein features compared to when HEAL solely used GCN in experiments. Additionally, the decrease in TAWFN’s evaluation metric values is more gradual as the homogeneity decreases, suggesting that the integration of protein language models and additional high-quality structures aids TAWFN in learning the relationship between structure and functional characteristics.

#### 3.7.2 TAWFN in the AFset test set

**Figure 6.**
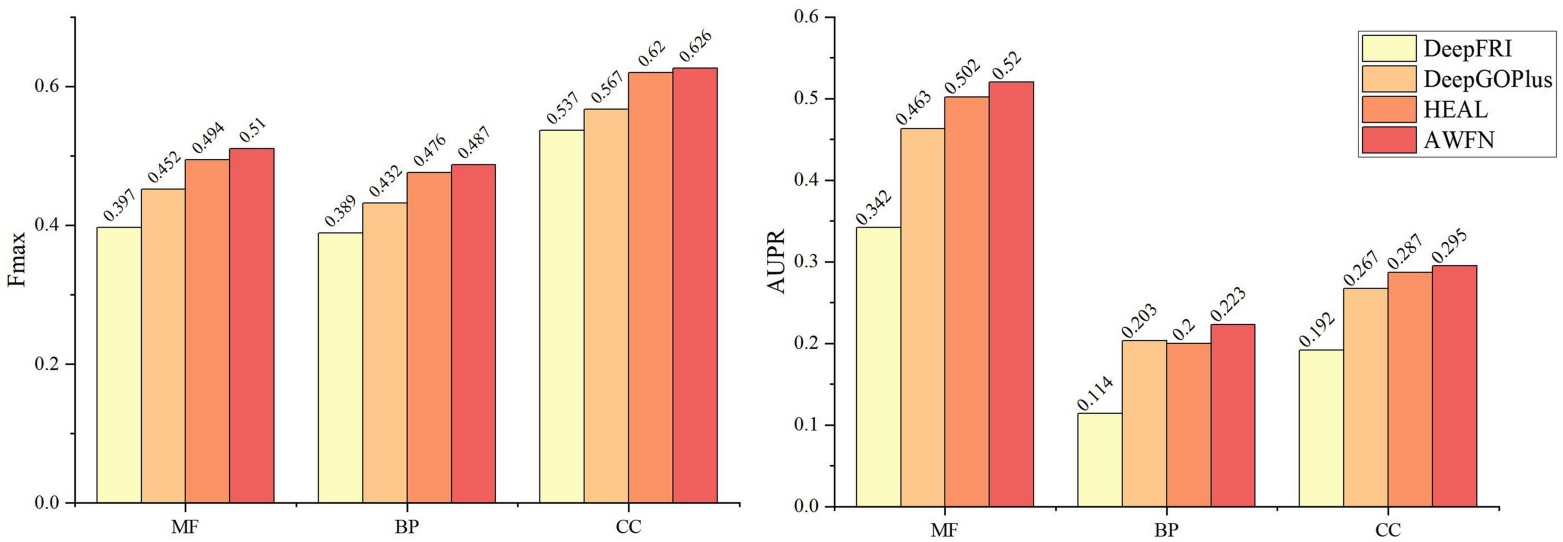
The AUPR and $F_{\max}$ of different methods on the AFset test set.

Experimental validation was conducted on the AFset test set, which comprises protein structures predicted by AlphaFold2. This evaluation is crucial as our method is intended for application on previously unseen protein structures to predict their functionalities. As depicted in Fig. 6, our approach outperforms methods solely based on GCNs [DeepFRI (Gligorijević *et al.* 2021) and HEAL (Gu *et al.* 2023)] and also achieves better results compared to the upgraded version of DeepGO (Kulmanov *et al.* 2018), DeepGOPlus (Kulmanov and Hoehndorf 2020). These findings underscore the effectiveness of combining MCNN and AGCN, enabling a more comprehensive learning of protein structure features, thereby capturing both structural and sequence characteristics for more accurate functional predictions.

## 4 Conclusion

This article proposes a novel method that combines multi-layer convolutional neural networks and adaptive graph convolutional neural networks, which has shown significant effectiveness in predicting protein structure functions. Compared to other methods, ours achieved better results. In our experiments, besides simple one-hot feature encoding, we also utilized protein language models to generate sequence features. The ablation experiments demonstrated that the performance of AGCN and MCNN individually was inferior to their combination, thus validating the effectiveness of the integration. Additionally, testing on protein structures with lower homology to the training set from the AlphaFold2 dataset also demonstrated stronger predictive capability, providing strong support for the practical application of our method.

By integrating MCNN and AGCN, we can capture protein structure features from different perspectives and scales. Introducing multi-head attention mechanisms in both MCNN and AGCN, the MCAM in MCNN effectively captures sequence features in protein structures, while the MHA in AGCN utilizes graph representation to capture relationships between protein structures from a global perspective. Moreover, the multi-scale feature extraction method allows us to comprehensively understand the complexity of protein structures, thereby improving the accuracy of protein function prediction. The structure of proteins is closely related to their function, and MCNN and AGCN can model protein structure and corresponding sequence features from both local and global perspectives, thereby better understanding the relationship between structure and function. The integration of MCNN and AGCN improves the accuracy and interpretability of protein function prediction, providing researchers with powerful tools to explore the relationship between protein structure and function. It also enables the fusion of cross-domain information, such as sequences, structures, and functional annotations. In future work, we aim to introduce more learnable features, utilize multi-view techniques, and predict novel protein structures.

## Supplementary Material

btae571_Supplementary_Data
