# Supplementary material for: TAWFN: a deep learning framework for protein function prediction
Source: Bioinformatics. 2024 Sep 23;40(10):btae571. doi: 10.1093/bioinformatics/btae571 (PMC11639667; doi:10.1093/bioinformatics/btae571)
Supplement: btae571_Supplementary_Data [file btae571_supplementary_data.docx]

Supplementary Information for

TAWFN: A Deep Learning Framework for Protein Function Prediction

**Evaluation metrics**

1. $F_{\max}$

Fmax is the maximum value of the F-measure (F1-score) across different thresholds. The F-measure is the harmonic mean of precision (Pc(t)) and recall (Rc(t)). In protein function prediction, precision represents the proportion of instances predicted as positive that are truly positive, while recall represents the proportion of all true positive instances that are predicted as positive.

The precision $pr_{i}\left( t \right)$and $rc_{i}\left( t \right)$ recall of the i-th protein under the threshold t are defined as follows:

$$\mathrm{pr}_{i}\left( t \right) =\frac{\left| T\cap P_{i}\left( t \right) \right|}{\left| P_{i}\left( t \right) \right|} (1)$$

$$\mathrm{rc}_{i}\left( t \right) =\frac{\left| T\cap P_{i}\left( t \right) \right|}{\left| T \right|} (2)$$

$$Pr\left( t \right)=\frac{1}{m\left( t \right)}\cdot\sum_{i=1}^{m\left( t \right)} pr_{i}\left( t \right) (3)$$

$$\mathrm{Rc}\left( t \right)=\frac{1}{n}\cdot\sum_{i=1}^{n} rc_{i}\left( t \right) (4)$$

$$F_{\max}=\max_{t}\left\{ \frac{2\cdot Pr\left( t \right)\cdot Rc\left( t \right)}{Pr\left( t \right)+Rc\left( t \right)} \right\} (5)$$

Where $m\left( t \right)$ refers to the number of proteins whose prediction probability of at least one functional label is greater than or equal to the threshold t, n and refers to the total number of proteins in the test set.

1. $S_{\min}$

Smin is a measure of the minimum semantic distance between the predicted results and the true labels. It is based on Information Content (IC), which is used to assess the similarity between the predicted set and the true set. Information Content (IC) is typically calculated based on the frequency of Gene Ontology (GO) annotations.

$$IC\left( l \right)=-log_{2}\left( P\left( l \right) \right) \left( 6 \right)$$

$$S_{false positive}\left( t \right)=\frac{1}{n}\sum_{i=1}^{n} \sum_{c\in T_{i}-P_{i}\left( t \right)} IC\left( l \right) \left( 7 \right)$$

$$S_{false negative}\left( t \right)=\frac{1}{n}\sum_{i=1}^{n} \sum_{c\in P_{i}\left( t \right)-T_{i}} IC\left( l \right) \left( 8 \right)$$

$$S_{\min}=\min_{t}\sqrt{{S_{false positive}\left( t \right)}^{2}+{S_{false negative}\left( t \right)}^{2}} \left( 9 \right)$$

1. AUPR

AUPR is the area under the Precision-Recall curve. The Precision-Recall curve shows the Precision and Recall values of a model at different thresholds. The larger the AUPR, the better the model performance. The calculation method typically uses the trapezoidal rule to approximate. This can be obtained by plotting the Precision-Recall curve and calculating the area under the curve.

Fig. S1. Ancestral graph of nuclear lumen (GO:0031981) in the CC category.

**Baseline methods**

BLAST (Altschul SF, Madden TL, Schäffer AA et al. (1997)) is a fundamental local alignment search tool that directly optimizes local similarity measures for alignments, such as the Maximum Segment Pair (MSP) score. It enables direct searches in DNA and protein sequence databases.

FunFams (Das S, Lee D, Sillitoe I et al. (2015)) is a classic method that has achieved good results in the CAFA competition. It tests sequences against the CATH HMM library. First, the test sequences are mapped to the most likely FunFam (i.e., the one with the highest HMM score). Then, the GO terms and EC numbers of that FunFam are transferred to the test sequence. The confidence score for each predicted GO term is calculated based on the annotation frequency of that GO term in the seed sequences of the FunFam.

DeepGO (Kulmanov M, Khan MA, Hoehndorf R et al. (2018)) combines CNN to learn sequence-level embeddings and integrates them with knowledge graph embeddings obtained from protein-protein interaction (PPI) networks. It utilizes the dependencies between GO classes as background information to construct a deep learning model.

DeepFRI (Gligorijević, V., Renfrew, P.D., Kosciolek, T. et al. (2021)) utilizes a graph convolutional network to predict protein functions by extracting features from protein structures and protein language models. DeepFRI demonstrates significant denoising capabilities and achieves excellent results in protein function prediction.

HEAL (Zhonghui Gu, Xiao Luo, Jiaxiao Chen et al. (2023)) builds upon DeepFRI by introducing a hierarchical graph transformer. It constructs super nodes to process protein structures and obtained sequence features through graph convolutional networks. Additionally, it expands the dataset. Currently, it is the most effective method for predicting protein functions based on protein structures. Our experiments are also conducted based on the HEAL dataset.

**Different numbers of heads in the multi-head attention mechanism in MCNN and the number of GCN layers in AGCN.**

Table S1. Performance of MCNN with different h on PDBset.

|  | AUPR(↑) | | | $F_{\max}$(↑) | | | | | $S_{\min}$(↓) | | |
| --- | --- | --- | --- | --- | --- | --- | --- | --- | --- | --- | --- |
|  | MF | BP | CC | | MF | BP | CC | MF | | BP | CC |
| h = 2 | 0.701 | 0.374 | 0.482 | | 0.752 | 0.627 | **0.696** | 0.338 | | 0.484 | **0.451** |
| h = 4 | **0.718** | **0.385** | **0.488** | | **0.762** | **0.628** | 0.693 | **0.326** | | **0.483** | 0.454 |
| h = 6 | 0.708 | 0.367 | 0.472 | | 0.754 | 0.617 | 0.69 | 0.333 | | 0.492 | 0.456 |
| h = 8 | 0.703 | 0.369 | 0.472 | | 0.751 | 0.621 | 0.695 | 0.338 | | 0.488 | 0.453 |

Table S2. Performance of AGCN with different n on PDBset.

|  | AUPR(↑) | | | $F_{\max}$(↑) | | | | | $S_{\min}$(↓) | | |
| --- | --- | --- | --- | --- | --- | --- | --- | --- | --- | --- | --- |
|  | MF | BP | CC | | MF | BP | CC | MF | | BP | CC |
| n = 1 | 0.715 | 0.361 | 0.471 | | 0.757 | 0.619 | 0.692 | 0.332 | | 0.494 | 0.457 |
| n = 2 | 0.714 | 0.369 | 0.47 | | 0.758 | 0.619 | **0.699** | 0.33 | | 0.493 | **0.453** |
| n = 3 | 0.718 | **0.385** | **0.488** | | **0.762** | **0.628** | 0.693 | **0.326** | | **0.483** | 0.454 |
| n = 4 | **0.72** | 0.36 | 0.472 | | 0.758 | 0.62 | 0.692 | 0.331 | | 0.494 | 0.454 |

**TAWFN performance at different thresholds**

Table S3. AUPR of TAWFN and other methods in the PDBset at different similarity thresholds.

| AUPR of MF ontology | | | | | |
| --- | --- | --- | --- | --- | --- |
| Model | <30% | <40% | <50% | <70% | <95% |
| DeepGO | 0.303 | 0.324 | 0.348 | 0.384 | 0.395 |
| DeepFRI | 0.426 | 0.447 | 0.461 | 0.489 | 0.503 |
| HEAL | 0.635 | 0.643 | 0.661 | 0.678 | 0.695 |
| TAWFN | **0.672** | **0.68** | **0.691** | **0.701** | **0.718** |
| AUPR of BP ontology | | | | | |
| Model | <30% | <40% | <50% | <70% | <95% |
| DeepGO | 0.138 | 0.132 | 0.156 | 0.172 | 0.184 |
| DeepFRI | 0.215 | 0.219 | 0.235 | 0.253 | 0.267 |
| HEAL | 0.300 | 0.294 | 0.310 | 0.324 | 0.346 |
| TAWFN | **0.342** | **0.342** | **0.353** | **0.371** | **0.385** |
| AUPR of CC ontology | | | | | |
| Model | <30% | <40% | <50% | <70% | <95% |
| DeepGO | 0.223 | 0.224 | 0.236 | 0.245 | 0.275 |
| DeepFRI | 0.247 | 0.248 | 0.254 | 0.256 | 0.287 |
| HEAL | 0.427 | 0.434 | 0.435 | 0.445 | 0.466 |
| TAWFN | **0.449** | **0.45** | **0.46** | **0.471** | **0.488** |

Table S4. $F_{\max}$of TAWFN and other methods in the PDBset at different similarity thresholds.

| $F_{\max}$of MF ontology | | | | | |
| --- | --- | --- | --- | --- | --- |
| Model | <30% | <40% | <50% | <70% | <95% |
| DeepGO | 0.483 | 0.504 | 0.525 | 0.559 | 0.572 |
| DeepFRI | 0.544 | 0.554 | 0.575 | 0.602 | 0.628 |
| HEAL | 0.698 | 0.703 | 0.720 | 0.736 | 0.748 |
| TAWFN | **0.716** | **0.725** | **0.735** | **0.753** | **0.762** |
| $F_{\max}$of BP ontology | | | | | |
| Model | <30% | <40% | <50% | <70% | <95% |
| DeepGO | 0.466 | 0.466 | 0.475 | 0.485 | 0.496 |
| DeepFRI | 0.501 | 0.512 | 0.517 | 0.533 | 0.542 |
| HEAL | 0.582 | 0.578 | 0.582 | 0.593 | 0.595 |
| TAWFN | **0.612** | **0.613** | **0.613** | **0.623** | **0.628** |
| $F_{\max}$of CC ontology | | | | | |
| Model | <30% | <40% | <50% | <70% | <95% |
| DeepGO | 0.583 | 0.584 | 0.585 | 0.589 | 0.595 |
| DeepFRI | 0.604 | 0.606 | 0.607 | 0.606 | 0.613 |
| HEAL | 0.684 | 0.683 | 0.684 | 0.685 | 0.687 |
| TAWFN | **0.695** | **0.692** | **0.692** | **0.69** | **0.693** |

Table S5. $S_{\min}$of TAWFN and other methods in the PDBset at different similarity thresholds.

| $S_{\min}$of MF ontology | | | | | |
| --- | --- | --- | --- | --- | --- |
| Model | <30% | <40% | <50% | <70% | <95% |
| DeepGO | 0.542 | 0.532 | 0.514 | 0.485 | 0.475 |
| DeepFRI | 0.505 | 0.500 | 0.485 | 0.453 | 0.436 |
| HEAL | 0.391 | 0.385 | 0.374 | 0.353 | 0.342 |
| TAWFN | **0.373** | **0.365** | **0.354** | **0.336** | **0.326** |
| $S_{\min}$of BP ontology | | | | | |
| Model | <30% | <40% | <50% | <70% | <95% |
| DeepGO | 0.596 | 0.595 | 0.589 | 0.575 | 0.573 |
| DeepFRI | 0.570 | 0.564 | 0.561 | 0.545 | 0.541 |
| HEAL | 0.524 | 0.526 | 0.521 | 0.514 | 0.512 |
| TAWFN | **0.494** | **0.494** | **0.495** | **0.486** | **0.483** |
| $S_{\min}$of CC ontology | | | | | |
| Model | <30% | <40% | <50% | <70% | <95% |
| DeepGO | 0.554 | 0.556 | 0.551 | 0.546 | 0.540 |
| DeepFRI | 0.533 | 0.527 | 0.523 | 0.525 | 0.524 |
| HEAL | 0.463 | 0.461 | 0.458 | 0.461 | 0.458 |
| TAWFN | **0.451** | **0.456** | **0.454** | **0.456** | **0.454** |

Table S6. The results of TAWFN and several other methods on the CAFA3 test dataset.

| Model | AUPR | | | Fmax | | |
| --- | --- | --- | --- | --- | --- | --- |
|  | MF | BP | CC | MF | BP | CC |
| DeepGO | 0.312 | 0.213 | 0.446 | 0.392 | 0.362 | 0.502 |
| DeepGOCNN | 0.402 | 0.213 | 0.523 | 0.411 | 0.388 | 0.582 |
| TALE | 0.485 | 0.258 | 0.649 | 0.548 | 0.398 | 0.654 |
| TransFun | 0.489 | 0.333 | 0.634 | 0.551 | 0.395 | **0.659** |
| MMSMAPlus | **0.559** | 0.470 | 0.601 | **0.595** | 0.535 | 0.622 |
| TAWFN | 0.497 | **0.613** | **0.722** | 0.535 | **0.539** | 0.651 |
